# Effects of Group Postural Rehabilitation in Patients with Parkinson’s Disease and Camptocormia: Preliminary Data from an Observational Pilot Study

**DOI:** 10.3390/bioengineering13101197

**Published:** 2026-10-15

**Authors:** Rossella Calciano, Paola Emilia Ferrara, Alberto Cutaia, Letizia Tarallo, Virginia Capolongo, Rosa Cioffi, Diego Ricciardi, Ludovica Tassi, Maria Rita Lo Monaco, Daniele Coraci, Gianpaolo Ronconi

**Affiliations:** 1 Department of Geriatrics, Orthopedics and Rheumatology, Fondazione Policlinico Universitario “Agostino Gemelli” IRCCS, L.go A. Gemelli 8, 00168 Rome, Italy; rossella.calciano@guest.policlinicogemelli.it (R.C.); paolaemilia.ferrara@policlinicogemelli.it (P.E.F.); diego.ricciardi@policlinicogemelli.it (D.R.); ludovica.tassi@guest.policlinicogemelli.it (L.T.); mariarita.lomonaco@policlinicogemelli.it (M.R.L.M.); gianpaolo.ronconi@policlinicogemelli.it (G.R.); 2 University Department of Geriatric and Orthopedic Sciences, Università Cattolica del Sacro Cuore, L.go F. Vito 1, 00168 Rome, Italy; letizia.tarallo@guest.policlinicogemelli.it (L.T.); vircap1995@gmail.com (V.C.); dr.cioffirosa@gmail.com (R.C.); 3 Geriatrics Unit, Fondazione Policlinico Universitario A. Gemelli IRCCS, 00168 Rome, Italy; 4 Section of Rehabilitation, Department of Neuroscience—DNS, University of Padova, 35122 Padova, Italy; daniele.coraci@unipd.it

**Keywords:** Parkinson’s disease, camptocormia, postural control, falls, postural exercise, stabilometric analysis, quality of life

## Abstract

**Background and Objectives:** Parkinson’s disease (PD) is frequently associated with postural instability, including camptocormia, an involuntary anterior flexion of the thoracolumbar spine. This retrospective observational pilot study evaluated the effects of a postural group rehabilitation protocol on the stabilometric parameters, depressive symptoms, and quality of life in PD patients with camptocormia, and whether gains were maintained during a subsequent two-month period after completion of supervised group rehabilitation, during which patients were provided with a general home-based exercise program. **Materials and Methods:** Forty patients with idiopathic PD (Hoehn and Yahr II–III) were assessed at baseline (T0), after two months of twice-weekly rehabilitation (T1). At T2, only stabilometric follow-up was performed after two months without group exercise for 38 patients. Outcomes included SPPB, Tinetti, Berg Balance Scale, Hamilton Depression Rating Scale, PDQ-39, occiput-to-wall distance and stabilometric analysis. **Results:** At T1, Hamilton scores decreased from 11.44 ± 4.59 to 3.33 ± 3.04 and PDQ-39 from 82.22 ± 22.36 to 69.72 ± 16.54 (both *p* < 0.001). Occiput-to-wall distance improved significantly in three of six positions. The EO/EC Perimeter Ratio decreased significantly from T0 to T1 (*p* = 0.016). Between T1 and T2, EO Perimeter continued improving (*p* = 0.010), whereas the Perimeter Ratio worsened (*p* = 0.046), and EC Ellipse area, though non-significant, increased rather than decreased. **Conclusions:** A two-month protocol was followed by apparent improvements in depression, quality of life, postural alignment, and selected stabilometric parameters. After discontinuation, some parameters appeared to improve while others seemed to worsen, suggesting these effects may not be sustained with uniformity. These preliminary findings should be confirmed with an expanded sample size in larger studies.

## 1. Introduction

Parkinson’s Disease (PD) is a chronic and progressive neurodegenerative disorder, and its cardinal motor symptoms are bradykinesia, rigidity and tremor. This condition results from the degeneration of dopaminergic neurons in the substantia nigra, compromising the proper modulation of movement through the basal ganglia [1]. Alongside these symptoms, gait and balance impairments commonly manifest in the intermediate-to-advanced stages of the disease; this leads to postural instability, freezing of gait, and loss of adaptability to external perturbations, increasing the risk of falls and reducing functional autonomy [2,3]. The literature underlines that the management of these motor functions requires various tools and different afferent inputs (visual, vestibular, and somatosensory) at both spinal and supraspinal levels [2,3].

Postural abnormalities are common in PD and may manifest as axial deformities, including camptocormia, Pisa syndrome, and antecollis. Among these, camptocormia is defined as an involuntary anterior flexion of the thoracolumbar spine that appears during standing and walking and is partially or completely reduced in the supine position. According to recent consensus criteria, camptocormia is identified by a thoracic flexion angle greater than 45° or lumbar flexion greater than 30° during upright posture and gait [4]. The prevalence of camptocormia in PD ranges from 3% to 18% [5], and its occurrence is associated with disease progression, axial rigidity, proprioceptive dysfunction, imbalance between trunk flexor and extensor muscles, and impaired sensory integration [5]. This condition is frequently associated with back pain and an increased risk of falls while standing or walking [6], and negatively impacts patients’ quality of life [7]. The abnormal forward displacement of the trunk alters the center of mass, compromises postural reactions, increases energy expenditure during gait, and further elevates fall risk. Patients frequently report musculoskeletal pain, fatigue, reduced walking capacity, and social embarrassment, all of which contribute to decreased quality of life and increased psychological burden.

Although pharmacological treatment with levodopa remains the cornerstone of PD management, its effectiveness on axial postural abnormalities is often limited, with significant clinical improvement observed mainly in patients with dystonic symptoms. In these cases, patients can benefit from deep brain stimulation (DBS) as a second-line therapeutic option [8,9,10], particularly when intervention occurs early after the onset of postural deformity [11]. Intramuscular injection of botulinum toxin [12,13] or lidocaine [14,15] may also improve camptocormia in patients with an associated dystonic component, although results across studies remain heterogeneous and inconsistent [16,17,18]. Consequently, rehabilitation has emerged as an essential complementary approach for managing postural and balance dysfunction in PD, encompassing traditional techniques and multifaceted interventions such as stretching and postural re-education [19]. Recent evidence suggests that rehabilitation may promote exercise-dependent neuroplasticity, improve motor learning, and enhance the sensory integration processes involved in postural control [20]. In particular, proprioceptive rehabilitation and trunk-focused exercise may improve balance performance by strengthening extensor musculature, optimizing body alignment, and reducing excessive visual dependence during postural tasks. Stabilometric analysis represents a valuable objective tool for quantifying postural sway and center-of-pressure displacement, allowing detailed assessment of balance strategies under eyes-open and eyes-closed conditions. Despite growing interest in rehabilitation strategies for PD, current evidence specifically addressing proprioceptive and postural rehabilitation in patients with camptocormia remains limited. We could not assess long-term effects at the end of the rehabilitation phase due to the pilot nature of the study. This is a clinically important gap: if the benefits of rehabilitation depend on continued practice, patients and clinicians need explicit guidance on whether—and which—gains are likely to regress once supervised treatment stops.

Therefore, the aim of the present study was twofold: first, to evaluate the effects of a two-month rehabilitation protocol—twice-weekly group sessions focused on trunk extensor strengthening, proprioceptive training, and postural re-education—on stabilometric parameters, postural alignment, depressive symptoms, and quality of life in patients with PD and camptocormia; and second, to determine whether these improvements were maintained during a subsequent two-month period following the end of the supervised group rehabilitation, during which patients did not attend further structured group sessions but were provided with a general home-based exercise program.

## 2. Materials and Methods

### 2.1. Study Design

This study was an observational, retrospective pilot study evaluating the effects of a rehabilitation protocol on postural alignment, balance, and quality of life in patients with idiopathic Parkinson’s disease (PD), with particular focus on camptocormia. The study was carried out in accordance with the Declaration of Helsinki, and the protocol was approved by the Ethics Committee of IRCCS Fondazione Policlinico Universitario A. Gemelli (prot. N 0016285/22, 11 May 2022, ID 4935).

### 2.2. Participants

Forty patients with idiopathic Parkinson’s disease (Hoehn and Yahr stage II–III) meeting the inclusion criteria completed the rehabilitation program at the Physical Medicine and Rehabilitation Ward of the IRCCS Fondazione Policlinico Universitario A. Gemelli, Rome. All participants were assessed by a physiatrist and engaged in a rehabilitation program in accordance with routine clinical practice. All participants provided written informed consent prior to inclusion in the study. Only stabilometric assessment was performed in 38 patients at T2, as the remaining two patients were unable to attend the assessment due to logistical and family-related difficulties associated with their lack of autonomy. Missing data were handled by excluding these participants from the analysis. For the Hamilton Depression Rating Scale, the PDQ-39 and the occiput-to-wall distance, complete data at both T0 and T1 were available for 36 of the 40 patients. The remaining four patients had missing T1 data for these measures, either because the questionnaires were not returned or were incomplete (*n* = 3), or because the measurements were not recorded in the clinical records (*n* = 1), and were therefore excluded from the corresponding paired analyses.

### 2.3. Inclusion Criteria

Participants were eligible if they met the following criteria: (i) diagnosis of Parkinson’s disease according to the UK Brain Bank of London criteria; (ii) Disease severity through Hoehn and Yahr stage II–III; (iii) absence of cognitive impairment (Mini-Mental State Examination, MMSE > 24/30); (iv) individualized pharmacological therapy tailored to their stage of Parkinson’s disease, as prescribed by a specialist neurologist, with pharmacological treatment personalized according to disease severity and clinical presentation, and each patient regularly followed on an individual basis by their referring neurologist; (v) written informed consent.

### 2.4. Exclusion Criteria

Exclusion criteria included: (i) atypical parkinsonism; (ii) poor pharmacological compensation; (iii) presence of other neurological disorders; (iv) neuromuscular diseases; (v) osteoarticular pathologies; and (vi) cognitive impairment (MMSE < 24).

### 2.5. Study Timeline

**Figure 1:**
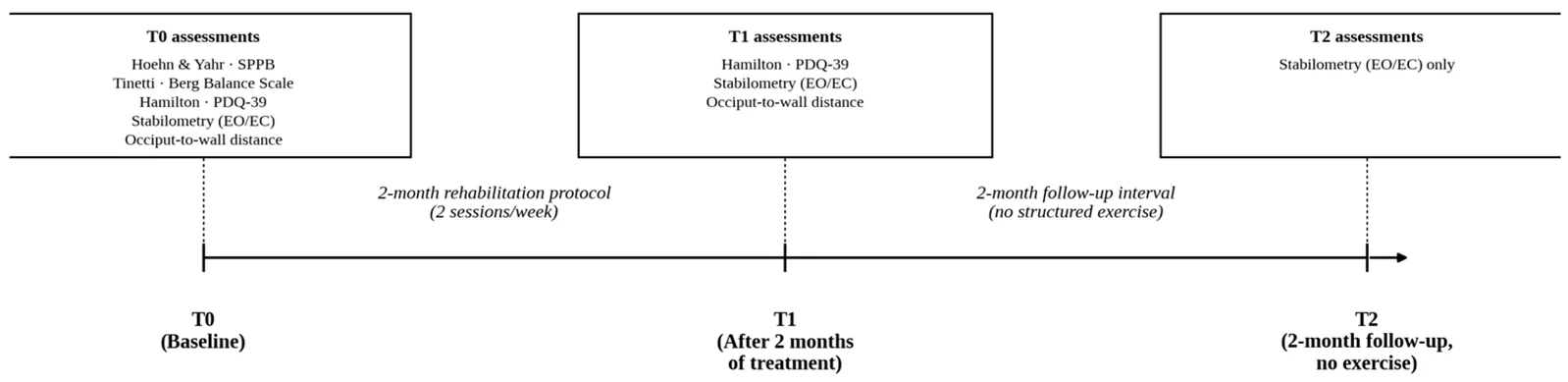
Study timeline and assessment schedule. T0: baseline; T1: after the two-month group rehabilitation protocol; T2: stabilometric-only follow-up.

The study followed a retrospective design comprising three assessment time points. Baseline assessment (T0) was performed prior to the start of treatment. Patients underwent two months of supervised rehabilitation, delivered twice weekly in group sessions; at the end of this period (T1), clinical and psychological scales (Hamilton Depression Rating Scale and PDQ-39), computerized stabilometry, and occiput-to-wall distance were repeated. A third assessment, limited to stabilometry, was performed two months after completion of the supervised group rehabilitation program (T2). During the T1–T2 period, patients did not participate in further structured group rehabilitation but were provided with a general home-based exercise program. Therefore, T2 represents a follow-up period after supervised group rehabilitation rather than a period of complete exercise withdrawal. Figure 1 illustrates the study timeline and the assessment schedule at each time point.

### 2.6. Rehabilitation Protocol

Participants completed a two-month physiotherapy program consisting of two 60-min group sessions per week (approximately 16 sessions in total). All sessions were conducted at 10:00 a.m., 1 h after the patients’ morning intake of levodopa, in medication-on state. All patients participated in the group-based rehabilitation program with good compliance. The rehabilitation protocol was designed to address postural impairment, axial rigidity, muscle weakness, and respiratory dysfunction associated with camptocormia. The program included strengthening exercises targeting the trunk extensors, scapular external rotators, interscapular muscles, quadriceps, and gluteal muscles, combined with stretching of the anterior muscle chains. Strengthening exercises were initially performed in 2 sets of 10 repetitions and progressively increased to 3 sets of 15 repetitions according to individual tolerance and performance. Also, respiratory exercises were included to promote thoracic expansion and improve ventilatory capacity, with the aim of reducing the worse and restrictive pattern associated with camptocormia. Exercises were performed in seated and standing positions and during gait training. The program included dynamic upper- and lower-limb coordination exercises, sustained postural holds, weight-shifting exercises, obstacle negotiation, visually guided walking tasks, and postural alignment exercises aimed at promoting trunk extension and head repositioning. Sustained postural positions were initially maintained for 10 s and progressively increased to 30 s. Weight-shifting and coordination exercises were initially performed for 10 repetitions and progressively increased to 15–20 repetitions, according to the patient’s functional capacity. During gait training, the duration and complexity of walking tasks were progressively increased, with the introduction of tasks such as obstacles, changes in direction, coordination and guided stepping (Table 1).

Exercise intensity, duration, number of repetitions, and task complexity were progressively increased. Progression was individually adapted according to each patient’s functional abilities and tolerance, while maintaining correct trunk and head alignment and adequate movement control. When appropriate, external support was gradually reduced to increase postural and motor demands. A physician was present during the sessions to ensure patient safety and to intervene if clinically indicated. Heart rate (HR), blood pressure (BP), and peripheral oxygen saturation (SpO_2_) could be assessed during the sessions when clinically necessary. No clinical events requiring medical intervention or monitoring of vital parameters occurred during any of the rehabilitation sessions. No adverse events related to the rehabilitation intervention were observed.

### 2.7. Outcome Measures

Patients were evaluated at baseline (T0) using the following tools:

- Lower-limb function and mobility were evaluated with the Short Physical Performance Battery (SPPB), which assesses gait, balance, and lower-extremity strength and has demonstrated strong psychometric properties and predictive validity for adverse outcomes [21].
- Postural stability and fall risk were assessed with the Tinetti Performance-Oriented Mobility Assessment [22] and the Berg Balance Scale, with established cut-off scores indicating mobility status and need for assistive devices [23].
- Depressive symptoms were evaluated with the Hamilton Depression Rating Scale, given the high prevalence of depression in PD [24].
- Quality of life was assessed with the PDQ-39, a disease-specific self-report measure [25].

Particularly, postural control was objectively evaluated using computerized stabilometric posturography (ProKin PK 254 P, TecnoBody Srl., Dalmine, Italy) under eyes-open (EO) and eyes-closed (EC) conditions.

The stabilometric assessment was performed using the ProKin PK 254 P computerized posturographic platform (TecnoBody Srl., Dalmine, Italy), which provides quantitative measurement of postural sway through the detection of center-of-pressure displacement during static standing tasks. The system allows objective recording of postural control under eyes-open and eyes-closed conditions and provides quantitative parameters such as ellipse area, sway path length (perimeter), and derived EO/EC ratios. From an engineering perspective, computerized stabilometry represents a sensor-based approach for the objective quantification of postural control, allowing the transformation of mechanical changes in body loading into measurable parameters that can be analyzed longitudinally. Center-of-pressure displacement was analyzed along the anteroposterior and mediolateral axes; the 95% confidence ellipse area and the sway path length (perimeter) were considered the primary stabilometric parameters, together with the area ratio and perimeter ratio between the eyes-open and eyes-closed conditions (EA/EC). Lower ellipse area and perimeter values indicate improved postural stability. Stabilometric assessment was performed in the standing position and lasted approximately 10 min from the patient’s entry into the assessment room, taking into account the slower movements and transfers associated with PD. Each recording lasted 30 s with eyes open and 30 s with eyes closed. Two trials were performed in total: one with eyes open and one with eyes closed. Two operators were involved in the stabilometric assessment.

Postural alignment was assessed using a modified occiput-to-wall distance (OWD) protocol, with foot positioning standardized at two distances from the wall (40 cm and 20 cm) to increase the postural demand of the task, in addition to a seated position. Measurements were taken both in a relaxed posture and during voluntary (forced) trunk extension. While the classic occiput/C7-to-wall distance is a validated measure of forward-flexed posture in PD [26,27] and has been used to quantify camptocormia severity [6], the graded-distance variant used here was adopted empirically within this rehabilitation protocol and has not, to our knowledge, been previously validated as a standardized instrument in the PD literature. OWD. The OWD measurement was always performed by the same operator, an experienced physiotherapist specialized in neurorehabilitation (D.R.), and three measurements were performed for each assessment.

Postural alignment and stabilometric analysis were evaluated at T1 at the end of the rehabilitation treatment. Stabilometric data were repeated at T2, after two months of treatment.

### 2.8. Statistical Analysis

Statistical analyses were performed using SPSS software (version 20.0; IBM Corp., Armonk, NY, USA). Continuous variables are expressed as mean ± standard deviation (SD). Comparisons between time points were performed using paired-samples *t*-tests. Paired analyses were conducted on the patients with available data at both time points (complete-case analysis); consequently, baseline (T0) means and SDs reported in result section refer to the subset of patients included in each paired comparison and may differ from those of the whole sample (*n* = 40) reported in Table 2. For each comparison, both the two-tailed *p*-value and, where applicable, a one-tailed *p*-value (calculated as two-tailed *p*/2) are reported. The directional hypotheses were defined a priori, before examination of the outcome data, based on the expected direction of improvement for each parameter. The one-tailed value was calculated only when the observed change was in the direction consistent with the corresponding pre-specified directional hypothesis; when the observed mean changed in the opposite direction, the one-tailed test was considered not applicable (n.a.) and only the two-tailed result was reported. Given the number of outcomes and statistical comparisons performed, findings based on one-tailed tests were interpreted cautiously, and two-tailed results were considered the primary reference for statistical significance. A *p*-value < 0.05 was considered statistically significant.

## 3. Results

### 3.1. Participants

A total of 40 patients with idiopathic Parkinson’s disease (16 men, 24 women; mean age 72.2 ± 8.0 years) completed the rehabilitation program and were included in the final analysis. Table 1 summarizes the baseline (T0) clinical characteristics of the participants. At baseline, patients showed moderate balance impairment and fall risk, as reflected by a mean Tinetti total score of 18.50 ± 4.25 (balance sub-score 9.50 ± 2.14; gait sub-score 9.00 ± 2.31) and a mean Berg Balance Scale score of 34.85 ± 5.24. Lower-limb functional performance, assessed with the SPPB, was reduced overall (total score 8.10 ± 2.02), with comparable impairment across the balance (2.40 ± 0.81), gait (3.65 ± 0.58), and chair sub-tests (2.05 ± 0.81). Depressive symptoms, measured with the Hamilton Depression Rating Scale, were on average within the mild range (11.85 ± 5.45), while quality of life, assessed with the PDQ-39, indicated a substantial disease-related burden (83.35 ± 22.96).

### 3.2. Stabilometric Parameters (T0, T1, T2)

Table 3 reports the stabilometric parameters at T0, T1, and T2, together with the significance of the paired comparisons. Under the eyes-open (EO) condition, the Ellipse area did not change significantly at any time point. Conversely, the Perimeter showed a significant reduction between T1 and T2 (two-tailed *p* = 0.010; one-tailed *p* = 0.005), indicating a delayed but measurable improvement in postural sway path length after the end of active treatment.

Under the eyes-closed (EC) condition, the Perimeter showed a reduction from T0 to T1 that reached significance only in the one-tailed test (*p* = 0.042), consistent with the pre-specified directional hypothesis, although the corresponding two-tailed value did not reach conventional significance (*p* = 0.083); the same pattern was observed between T0 and T2 (one-tailed *p* = 0.033; two-tailed *p* = 0.065). The EC Ellipse area did not differ significantly between any pair of time points; in particular, its mean value increased—rather than decreased—between T0 and T2, a change in the direction opposite to that expected with continued improvement.

The EO/EC Perimeter Ratio decreased significantly from T0 to T1 (two-tailed *p* = 0.016; one-tailed *p* = 0.008), indicating a relative improvement in eyes-closed postural control during active treatment. However, this ratio increased again between T1 and T2 (two-tailed *p* = 0.046), a change in the opposite direction to the treatment hypothesis, indicating only partial maintenance of this parameter once supervised rehabilitation ended. The EO/EC Area Ratio did not change significantly at any time point and increased progressively across all three assessments.

### 3.3. Occiput-to-Wall Distance (OWD) (T0–T1)

Table 4 reports occiput-to-wall distance at T0 and T1 across the six assessment positions in the 36 patients with data available at both time points. Significant reductions, consistent with improved postural alignment, were observed in the seated position (relaxed: *p* < 0.001; forced extension: *p* < 0.001) and in the standing position with feet 20 cm from the wall in the relaxed condition (*p* < 0.001). No significant change was observed in the standing position with feet 40 cm from the wall (relaxed or forced) or in the forced standing position at 20 cm.

### 3.4. Depression and Quality of Life (T0–T1)

Table 5 reports Hamilton Depression Rating Scale and PDQ-39 scores at T0 and T1. The analysis included the 36 patients with data available at both time points (T1 data were missing for four patients); for this reason, the T0 values in Table 4 (Hamilton Depression Rating Scale 11.44 ± 4.59; PDQ-39 82.22 ± 22.36) differ from those of the whole sample of 40 patients reported in Table 1 (11.85 ± 5.45 and 83.35 ± 22.96, respectively), which refer to the same T0 assessment. Both outcomes improved significantly: Hamilton scores decreased from 11.44 ± 4.59 to 3.33 ± 3.04 (*p* < 0.001), and PDQ-39 scores decreased from 82.22 ± 22.36 to 69.72 ± 16.54 (*p* < 0.001), indicating an improvement in quality of life. Neither scale was reassessed at T2, so their status during the post-rehabilitation follow-up period, excluding these participants, could not be evaluated in this study (see Section 4.4 Limitations).

### 3.5. Summary of Significant Findings

**Figure 2:**
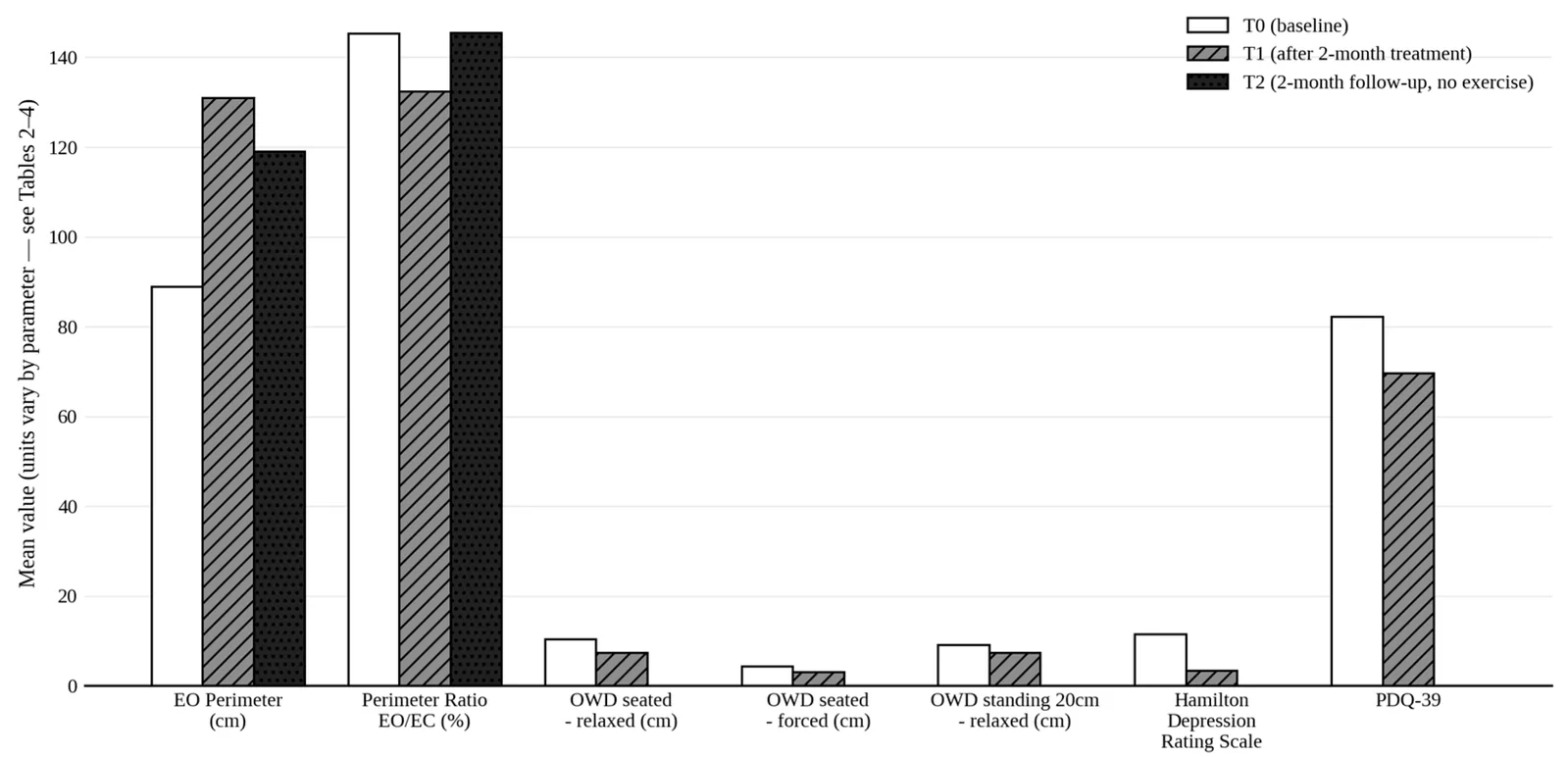
Mean values at T0, T1, and T2 (where available) for all parameters showing statistically significant change (two-tailed *p* < 0.05) in at least one paired comparison. Units differ across parameters (mm, cm, %, or scale points); see Table 2, Table 3 and Table 4 for details. Bars are omitted where a given time point was not significant or not assessed.

Figure 2 summarizes, in a grouped bar chart, all parameters that reached statistical significance (two-tailed *p* < 0.05) in at least one paired comparison: EO Perimeter (T1–T2), EO/EC Perimeter Ratio (T0–T1 and T1–T2), occiput-to-wall distance in the seated (relaxed and forced) and standing 20 cm (relaxed) positions, Hamilton Depression Rating Scale, and PDQ-39. Taken together, these findings indicate that the rehabilitation protocol produced measurable improvements in postural alignment, selected stabilometric parameters, depressive symptoms, and quality of life. However, the pattern observed between T1 and T2 was not uniform: while the eyes-open Perimeter continued to improve after treatment discontinuation, the EO/EC Perimeter Ratio worsened, and the EC Ellipse area trended toward worsening without reaching significance, suggesting that some, but not all, of the gains achieved during active treatment were maintained during the unsupervised follow-up period.

## 4. Discussion

This paper evaluated the effects of a two-month personalized rehabilitation protocol on stabilometric parameters, postural alignment, depressive symptoms, and quality of life in patients with PD and camptocormia. In addition, we highlight whether these improvements were maintained during a subsequent two-month period without exercises and rehabilitation.

### 4.1. Influence on Stabilometric Parameters and Postural Alignment

Despite pilot results, our rehabilitation protocol produced improvements in occiput-to-wall distance in the seated position and in the standing position at 20 cm from the wall and a significant reduction in the EO/EC Perimeter Ratio; these findings align with previous literature on postural and proprioceptive rehabilitation in PD [19,20]. Camptocormia represents a type of postural axial alteration of PD, being associated with impaired postural alignment, balance, gait, and functional independence; it is a condition poorly responsive to pharmacological treatment. Current evidence supports a multidisciplinary approach including physiotherapy and postural rehabilitation, although the available literature remains limited and heterogeneous [28]. In this context, the improvements in postural alignment observed in our study are clinically relevant, suggesting that an individualized rehabilitation program may contribute to improving axial control in patients with PD and camptocormia [5,29]. One of the most clinically relevant findings of this study concerns the changes observed after treatment discontinuation. Between T1 and T2, during the two-month period without structured exercise, the pattern was heterogeneous. The EO Perimeter had improvement, suggesting that postural control acquired may persist; this hypothesis reflects the process of motor learning and postural control acquired during rehabilitation that may persist after treatment [30].

In the literature, similar conditions of balance improvements after the end of rehabilitation have been reported in patients with PD, with benefits maintained from one to several months after balance-oriented interventions [31]. Wong Yu et al., in particular, underline the effects of a multidimensional balance training program on gait of PD patients with a longer follow-up of 12 months [32]. These findings are consistent with Smania et al., who reported significant improvements in postural instability after balance training, with most benefits maintained at one-month follow-up. Similarly, in our study, EO Perimeter continued to improve after treatment discontinuation.

However, the worsening of the EO/EC Perimeter Ratio and the trend toward increased EC Ellipse Area suggest that not all postural control components were maintained during the two-month period following the supervised group rehabilitation, supporting the importance of continued rehabilitation to preserve sensory-dependent balance [33]. Conversely, the EO/EC Perimeter Ratio, which had significantly improved during active treatment, worsened during the post-rehabilitation follow-up period, while the EC Ellipse Area showed a non-significant worsening trend. This different behavior among stabilometric parameters may indicate that sensory-dependent components of postural control are more susceptible to treatment discontinuation. Eyes-closed testing reduces visual input and therefore increases the relative contribution of non-visual sensory information, including somatosensory and vestibular inputs. Nevertheless, as reported in the literature, visual input increases the information provided by the vestibular and proprioceptive centre of control, so rehabilitation may improve the proprioceptive pathway in order to improve balance and postural control [34,35]. From an engineering perspective, the use of computerized stabilometry also highlights the potential role of sensor-based technologies in the objective assessment of postural control. In addition to currently available commercial posturographic systems, future developments may include the integration of low-cost sensing technologies based on innovative materials, potentially allowing more accessible and portable solutions for the quantitative assessment of postural parameters. In this context, resonant force sensors based on ionic polymer–metal composites have been proposed as an alternative sensing technology with potential applications in force measurement [36]. Although such technologies were not used in the present study, their development may represent an interesting direction for future research aimed at integrating low-cost sensor systems into rehabilitation assessment and monitoring.

Our pilot findings, which cover only the period following the rehabilitation group, should be viewed in light of the heterogeneous evidence on the rehabilitation effects in PD, and no inference can be drawn from our data regarding long-term outcomes. Some studies have reported retention of balance benefits after treatment cessation [33,37], whereas others indicate that these effects tend to decline without continued training, highlighting the potential importance of ongoing physical therapy in PD [38,39]. A recent meta-analysis similarly reported benefits of physiotherapy on balance and motor function during follow-up, although with considerable heterogeneity between interventions and outcome measures [40]. Nevertheless, it may be hypothesized that some motor strategies persist through learning and consolidation, whereas proprioceptive and sensory-motor components require continued stimulation. This interpretation is speculative, but it would be compatible with the view of rehabilitation in PD as a continuous process, combining supervised treatment with individualized maintenance exercise, possibly with caregiver support, as suggested by the 2024 International Consensus Statement by Goldman and colleagues [41]. Depressive symptoms and quality of life also showed improvements, in line with previous evidence. Paolucci et al. described an association between motor rehabilitation and psychological well-being in PD, suggesting that as patients regain functional and postural competence, their perceived disease burden and depressive symptoms may decrease [20]. However, given the single-arm design of our study, we cannot establish whether these changes were attributable to the rehabilitation program.

### 4.2. Depressive Symptoms and Quality of Life

Another important point is the evaluation of depressive symptoms and the reduction in quality of life experienced by these patients, related to the neurodegenerative condition. In our protocols, the Hamilton Depression Rating Scale and PDQ-39 were assessed only at T0 and T1 and were not reassessed at T2, and this could be a limitation. Therefore, although the active rehabilitation phase was associated with significant improvements in both outcomes, this study cannot determine whether these benefits were maintained, reduced, or lost during the free-exercise periods. Current evidence in the literature underlines the positive effects of exercise and training in PD, although future studies may need to focus on determining the possible long-term sustainability of these effects [42,43].

### 4.3. Rehabilitation Program Between T1 and T2

In a previous work [44], our research group investigated focal muscle vibration combined with standardized exercise, demonstrating short-term improvements in SPPB, Tinetti score, and Romberg Quotient and supporting the potential role of proprioceptive-oriented interventions in improving postural control. In the present paper, a group rehabilitation protocol was proposed, as described in detail in the dedicated section. At the end of the group rehabilitation program and before the stabilometric assessment at T2, patients were also provided with a general home-based exercise program aimed at maintaining physical activity and supporting the benefits achieved during the supervised intervention. This program included respiratory exercises, stretching exercises involving the four limbs, and sit-to-stand exercises. Patients were encouraged to perform these exercises regularly at home, under the supervision of a caregiver to ensure safety and security. They were also instructed to record any adverse events, pain or difficulties in a dedicated diary.

This was a retrospective, single-arm study without a control group, so the observed improvements should be interpreted as associations, not as evidence that the rehabilitation protocol caused them. Several alternative explanations cannot be excluded, including the natural course of PD, spontaneous fluctuations of camptocormia, regression to the mean, seasonal variability, placebo and practice effects, and the greater clinical attention and social interaction of a group-based program (Hawthorne effect).

### 4.4. Limitations

This study has several limitations. First, its retrospective, single-arm design and the absence of a control group do not allow causal inference regarding the effects of the rehabilitation intervention. Therefore, the changes observed over time cannot be attributed to the group rehabilitation program: spontaneous fluctuations, disease progression, medication effects, and other factors may be considered. Second, the sample size was small (*n* = 40 at baseline, *n* = 38 for most stabilometric comparisons, and *n* = 36 for the Hamilton Depression Rating Scale, PDQ-39 and occiput-to-wall distance comparisons), and two patients were lost at T2, which may have limited the statistical power to detect smaller effects, particularly in the T1–T2 and T0–T2 comparisons. In addition, complete T0–T1 data for the Hamilton Depression Rating Scale, the PDQ-39 and the occiput-to-wall distance were available for only 36 of the 40 patients, as four patients had missing T1 data for these measures. Since a complete-case analysis was performed, this further reduced the sample size for these comparisons, and we cannot exclude that the results were affected by selection bias if the excluded patients differed from those analysed. The high variability observed in some stabilometric parameters may have further contributed to this limitation. Third, clinical and psychological scales, including the Hamilton Depression Rating Scale and PDQ-39, were not repeated at T2. It was not possible to determine whether the improvements observed after the rehabilitation group program were maintained over time in terms of depressive symptoms and quality of life. Furthermore, the occiput-to-wall distance protocol used in this study was not validated and was adopted empirically. This limits the interpretation of changes in postural assessment. Another important limitation is the lack of detailed information regarding the individual levodopa dosage and other concomitant pharmacological treatments. Although rehabilitation sessions were performed 1 h after the usual morning levodopa intake, individual differences in levodopa dosage, treatment regimens, and concomitant other drugs were not reported. Therefore, the potential influence of dopaminergic therapy and other pharmacological treatments on stabilometric and clinical outcomes could not be adequately assessed. Nevertheless, the high variability observed for EO perimeter at T1 should be considered a limitation of the present study, as the distribution of this parameter and the potential influence of extreme observations were not specifically assessed. A further limitation of the present study is that effect sizes and 95% confidence intervals were not included in the original statistical analysis, and validated thresholds for clinically meaningful changes were not available for all of the stabilometric parameters assessed. Therefore, the magnitude and clinical relevance of some of the observed changes should be interpreted with caution. The relatively large number of outcomes and statistical comparisons represents a further limitation, as no formal adjustment for multiple comparisons was applied. Therefore, the possibility of an increased risk of type I error and false-positive findings cannot be excluded, and individual statistically significant results should be interpreted with caution. Finally, another important limitation regards the home-based exercise program: adherence to these exercises was not objectively monitored and we cannot establish their effects.

## 5. Conclusions

The present findings are preliminary and should be considered only as hypotheses. In our cohort of patients with PD and camptocormia, participation in a group-based rehabilitation program was accompanied by changes in postural alignment, selected stabilometric parameters, depressive symptoms, and quality of life. However, the retrospective single-arm design and the lack of a control group preclude any causal inference, and non-specific factors such as Hawthorne, placebo, and practice effects may have contributed to the observed changes. Accordingly, we cannot conclude that the rehabilitation program was responsible for these changes. At T2, following the end of the supervised program and during a period in which patients were given a general home-based exercise program, changes in stabilometric parameters were heterogeneous: some parameters appeared to improve further, whereas others worsened. This may indicate that different domains of postural control follow distinct trajectories after supervised rehabilitation. Because adherence to the home-based program was not monitored, no conclusions can be drawn about the contribution of unsupervised exercise to these results. Additional limitations should be acknowledged. Detailed information on individual levodopa dosage and concomitant pharmacological treatments was unavailable, so medication-related effects, including on/off fluctuations and possible therapeutic adjustments during follow-up, could not be adequately accounted for. Moreover, the small sample size and the limited number of assessment time points restrict the robustness and generalizability of the findings, and the durability of any observed effects remains uncertain.

Future prospective controlled studies with larger samples are needed to confirm or refute these preliminary observations. Such studies should include standardized documentation of pharmacological treatment, repeated clinical and psychological assessments, longer follow-up, and objective monitoring of adherence to home-based exercise (for example, wearable sensors or structured logs). Only such designs will make it possible to establish whether structured rehabilitation is effective in camptocormia associated with PD, how long its effects persist, and what role continued exercise plays after supervised treatment.

## Figures and Tables

**Table 1:** Description of the group physiotherapy rehabilitation protocol.

Rehabilitation Domain	Main Exercises	Position	Duration/Repetitions	Progression
Trunk extensor strengthening	Active trunk extension and postural correction exercises	Sitting and standing	Repeated exercises according to individual tolerance	Progressive increase in repetitions and range of movement, with gradual reduction in external support
Scapular and interscapular muscle strengthening	Scapular retraction and external rotation exercises	Sitting and standing	Repeated sets according to individual tolerance	Progressive increase in repetitions and exercise difficulty
Quadriceps and gluteal strengthening	Lower-limb strengthening exercises	Sitting and standing	Repeated sets according to individual tolerance	Progressive increase in repetitions and resistance, when appropriate
Stretching of anterior muscle chains	Stretching exercises targeting the anterior trunk and lower-limb muscle chains	Sitting and standing	Sustained positions, progressively increased according to tolerance	Holding time progressively increased up to 30 s
Respiratory exercises	Thoracic expansion and controlled breathing exercises	Mainly sitting	Repeated respiratory cycles	Progressive increase in repetitions and emphasis on thoracic expansion
Postural alignment	Trunk extension, head repositioning, and maintenance of upright alignment	Sitting and standing	Sustained postural holds initially maintained for 10 s	Progressive increase in holding time up to 30 s
Weight shifting	Anterior–posterior and lateral weight-shifting exercises	Standing	Repeated trials according to individual tolerance	Progressive increase in excursion, repetitions, and reduction in external support
Upper- and lower-limb coordination	Dynamic, alternating and combined limb movements	Sitting and standing	Repeated sequences	Progressive increase in repetitions, movement complexity, and speed
Gait training	Walking, changes in direction, and visually guided walking tasks	Walking	Repeated walking trials	Progressive increase in duration and complexity
Obstacle negotiation	Stepping over and around obstacles	Walking	Repeated trials	Progressive increase in obstacle complexity and reduction in external cues

**Table 2:** Characteristics of the patients at baseline (T0). All 40 patients included.

Characteristics	Group (*n* = 40)
Sex (M/F)	16/24
Age (years)	72.2 ± 8.0
Tinetti—Balance	9.50 ± 2.14
Tinetti—Gait	9.00 ± 2.31
Tinetti—Total	18.50 ± 4.25
SPPB—Balance	2.40 ± 0.81
SPPB—Gait	3.65 ± 0.58
SPPB—Chair (sit-to-stand)	2.05 ± 0.81
SPPB—Total	8.10 ± 2.02
Berg Balance Scale	34.85 ± 5.24
Hamilton Depression Rating Scale	11.85 ± 5.45
PDQ-39	83.35 ± 22.96 ^1^

^1^ Values are expressed as mean ± SD.

**Table 3:** Stabilometric parameters at T0, T1, and T2 (Mean ± SD) and significance of paired comparisons.

Parameter	T0 (Mean ± SD) *n* = 40	T1 (Mean ± SD) *n* = 38	T2 (Mean ± SD) *n* = 38	*p*-Value (1-Tailed/2-Tailed) T0–T1	*p*-Value (1-Tailed/2-Tailed) T1–T2|T0–T2
EO Ellipse (mm^2^)	1233.80 ± 897.79	1217.05 ± 940.22	1381.32 ± 1277.91	0.456/0.911	n.a. ^1^/0.107|n.a. ^1^/0.126
EC Ellipse (mm^2^)	2141.35 ± 1893.83	2060.00 ± 1878.00	2421.26 ± 2256.84	0.306/0.613	n.a. ^1^/0.261|n.a. ^1^/0.401 ^2^
EO Perimeter (mm)	888.65 ± 463.61	1309.68 ± 1626.64	1190.58 ± 1632.10	n.a. ^1^/0.118	0.005 */0.010 *|n.a. ^1^/0.266
EC Perimeter (mm)	1293.90 ± 774.94	1265.00 ± 792.82	1183.05 ± 641.44	0.042 */0.083	0.104/0.208|0.033 */0.065
EO/EC Area Ratio (%)	171.95 ± 66.97	183.26 ± 98.34	194.53 ± 136.63	n.a. ^1^/0.393	n.a. ^1^/0.571|n.a. ^1^/0.321
EO/EC Perimeter Ratio (%)	145.30 ± 44.09	132.47 ± 31.13	145.47 ± 39.33	0.008 */0.016 *	n.a. ^1^/0.046 *|n.a. ^1^/0.871

* *p* < 0.05. Values in each significance column are reported as one-tailed/two-tailed *p*-values. ^1^ n.a.: the one-tailed test was not applicable because the observed mean changed in the direction opposite to the a priori hypothesis of improvement. ^2^ The T0–T2 comparison for Ellipse EC showed a numerical increase (worsening) of the mean value that did not reach statistical significance; this finding should be interpreted with caution (see Section 4. Discussion).

**Table 4:** Occiput-to-wall distance (OWD) at T0 and T1 (Mean ± SD) and significance. Analyses were performed on the 36 patients with data available at both T0 and T1; T0 values therefore refer to this subset.

Position	T0 (Mean ± SD)	T1 (Mean ± SD)	*p*-Value (1-Tailed)	*p*-Value (2-Tailed)
Seated—relaxed	10.39 ± 5.38	7.39 ± 3.92	0.000 *	0.000 *
Seated—forced (extension)	4.33 ± 2.81	3.04 ± 2.86	0.000 *	0.000 *
Standing 40 cm from wall—relaxed	9.22 ± 6.03	13.31 ± 26.28	n.a. ^1^	0.331
Standing 40 cm from wall—forced	4.67 ± 4.37	4.89 ± 4.38	n.a. ^1^	0.422
Standing 20 cm from wall—relaxed	9.06 ± 5.78	7.39 ± 5.90	0.000 *	0.000 *
Standing 20 cm from wall—forced	5.39 ± 4.97	5.11 ± 4.51	0.194	0.388

* *p* < 0.05. ^1^ n.a.: one-tailed test not applicable (mean changed in the direction opposite to the hypothesis of improvement).

**Table 5:** PDQ-39 and Hamilton Depression Rating Scale at T0 and T1 (Mean ± SD) and significance (*n* = 36 patients with data available at both T0 and T1; T0 values therefore differ from those of the whole sample of 40 patients in Table 1).

Scale	T0 (Mean ± SD)	T1 (Mean ± SD)	*p*-Value (1-Tailed)	*p*-Value (2-Tailed)
Hamilton Depression Rating Scale	11.44 ± 4.59	3.33 ± 3.04	0.000 *	0.000 *
PDQ-39	82.22 ± 22.36	69.72 ± 16.54	0.000 *	0.000 *

* *p* < 0.05.

## Data Availability

Data associated with the paper are not publicly available but are available from the corresponding author on reasonable request.
